# Rare Presentation of Cervical Ganglioneuroma: A Case Report

**DOI:** 10.7759/cureus.88427

**Published:** 2025-07-21

**Authors:** Mohammed Bassami, Aymene Benmansour, Hicham Mimouni, Rajae Borki, Ilham Rkain

**Affiliations:** 1 Department of Otorhinolaryngology, Head and Neck Surgery, University Hospital Mohammed VI, Faculty of Medicine and Pharmacy, Abdelmalek Essaadi University, Tangier, MAR

**Keywords:** benign tumors, cervical ganglioneuroma, cervical mass, head and neck tumor, neurogenic tumor

## Abstract

Ganglioneuromas are rare, benign neoplasms of autonomic origin that represent the most differentiated end of the spectrum of peripheral neuroblastic tumors. Most commonly found in the posterior mediastinum and retroperitoneum, cervical involvement is distinctly uncommon. These tumors usually remain asymptomatic until they exert pressure on surrounding structures. We report the case of a 19-year-old boy who presented with a progressive right-sided cervical mass and contralateral vocal cord paralysis. Imaging revealed a well-circumscribed, encapsulated lesion medial to the carotid space. Surgical excision was performed, and histopathological analysis confirmed the diagnosis of ganglioneuroma. This case is notable not only for the tumor's rare cervical location but also for its unusual presentation with contralateral vocal cord paralysis. It underscores the importance of considering ganglioneuroma in the differential diagnosis of pediatric neck masses and highlights the critical role of histopathological confirmation in guiding management.

## Introduction

Ganglioneuromas (GN) are rare, benign neoplasms of neuroblastic origin that arise from the autonomic nervous system [1]. Their incidence is estimated at approximately one in 1,000,000 individuals, making them an uncommon clinical entity. These tumors occur more frequently in the pediatric population and young adults, with approximately 60% of cases diagnosed in patients under 20 years of age, with a slight female predominance [2]. Ganglioneuromas are most frequently found in the posterior mediastinum and retroperitoneum with the involvement of the cervical region being very uncommon. When present in the neck, they may manifest as a swelling, pain, or symptoms related to compression of the upper aerodigestive tract [3]. Histologically, GN represent the most differentiated form of peripheral neuroblastic tumors, characterized by the presence of mature ganglion cells within a Schwannian stroma. Although considered benign, cases of local recurrence and rare malignant transformation have been reported [4].

We present here the case of a 19-year-old boy with a right-sided cervical mass diagnosed as a cervical ganglioneuroma, along with a review of the relevant literature. This case underscores the importance of a thorough differential diagnosis, emphasizing the need to consider ganglioneuroma as a potential cause of cervical masses.

## Case presentation

**Figure 1:**
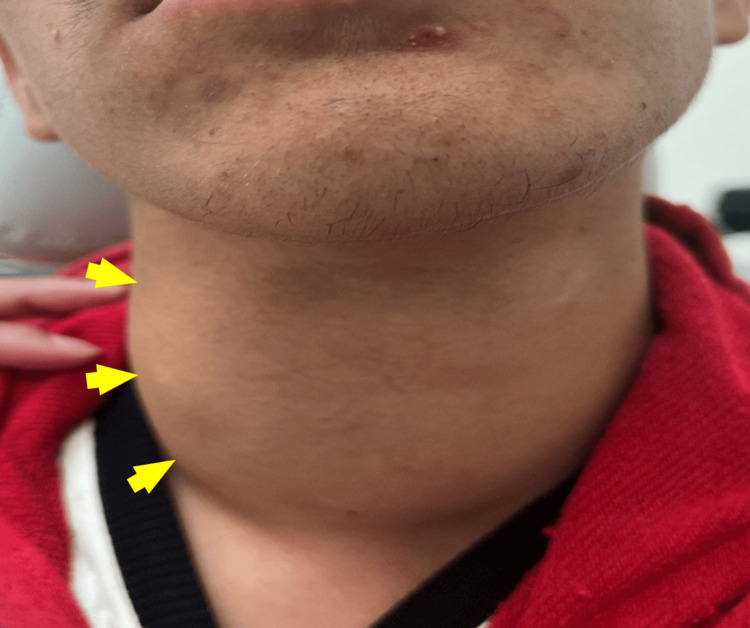
Anterior view showing right anterolateral neck mass (yellow arrows).

A 19-year-old boy with no significant past medical history was admitted for evaluation of a right cervical swelling that had been progressively enlarging over 18 months. There was no history of fever, and his general condition remained preserved throughout. On clinical examination, the patient was alert, hemodynamically and respiratory stable. A firm, non-tender right cervical mass was noted (Figure 1), accompanied by dysphonia that had been present for four months, as well as palpable left cervical lymphadenopathy.

Initial laboratory investigations were unremarkable, showing a normal complete blood count, normal liver and renal function, and no elevation of inflammatory markers (Table 1).

**Table 1:** Laboratory Results of Our Patient. AST: Aspartate transferase; ALT: alanine transaminase; SGOT: serum glutamic-oxaloacetic transaminase; SGPT: serum glutamic-pyruvic transaminase; VMA: vanillylmandelic acid; HVA: homovanillic acid.

Parameter	Patient Value	Normal Range
Hemoglobin (g/dL)	14.2	13.0-17.0
White Blood Cell Count (×10⁹/L)	6.5	4.0-10.0
Platelet Count (×10⁹/L)	250	150-400
Urea (mg/dL)	28	10-50
Creatinine (mg/dL)	0.8	0.6-1.2
AST / SGOT (U/L)	22	10-40
ALT / SGPT (U/L)	25	7-56
Total Bilirubin (mg/dL)	0.7	0.2-1.2
Direct Bilirubin (mg/dL)	0.2	0.0-0.4
Alkaline Phosphatase (U/L)	85	40-130
C-Reactive Protein (mg/L)	3.0	<5.0
VMA (mg/24h urine)	3.2	<6.8
HVA (mg/24h urine)	4.1	< 8.0

**Figure 2:**
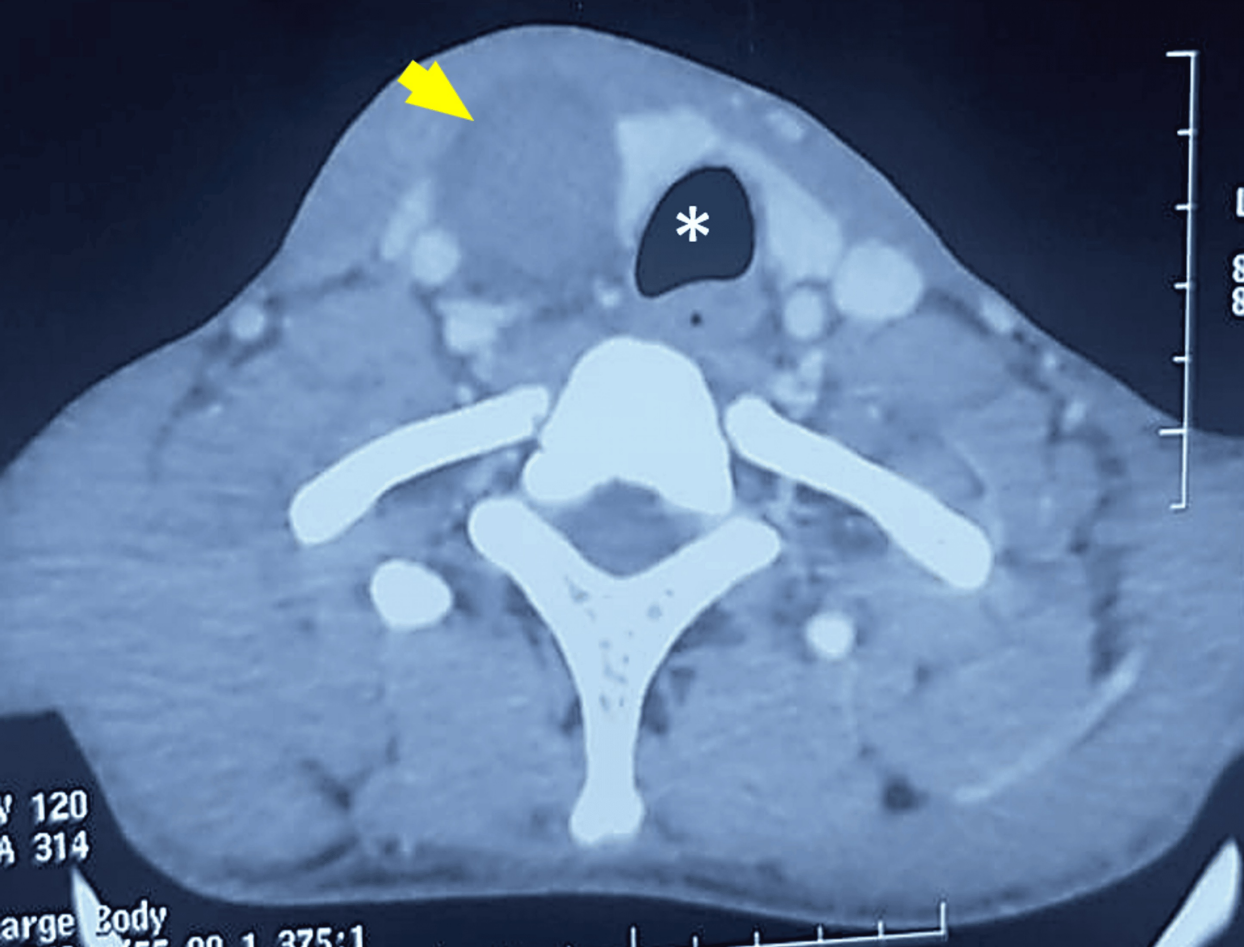
Axial CT image showing a right laterocervical mass (yellow arrow) exerting a mass effect on the trachea (asterisk), at the level of T1.

Nasofibroscopy revealed left vocal cord paralysis, contralateral to the mass. This likely results from compression or injury to the opposite recurrent laryngeal nerve caused by the mass effect. Cervical computed tomography (CT) demonstrated a large, well-defined, deep right latero-cervical mass extending from the level of C5 to T1, medial to the ipsilateral carotid space. The lesion was oval-shaped, thin-walled, and mildly heterogeneous, showing discrete contrast enhancement. It measured 6.5×4.2×3.5 cm and exerted a mass effect on the laryngopharyngeal structures, displacing them to the left and compressing the right thyroid lobe without a visible cleavage plane (Figure 2).

**Figure 3:**
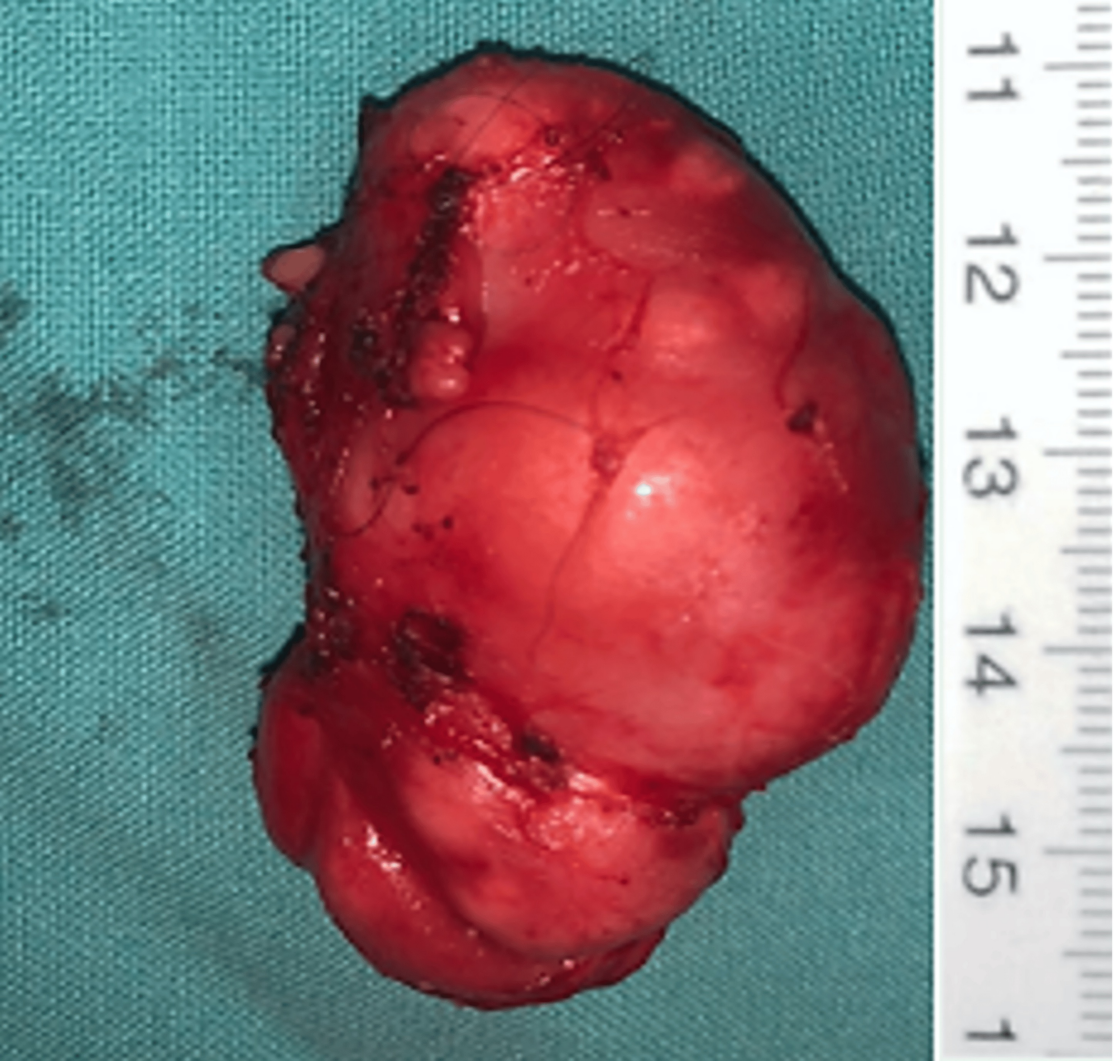
Gross specimen of the excised mass revealing a smooth, well-encapsulated tumor.

**Figure 4:**
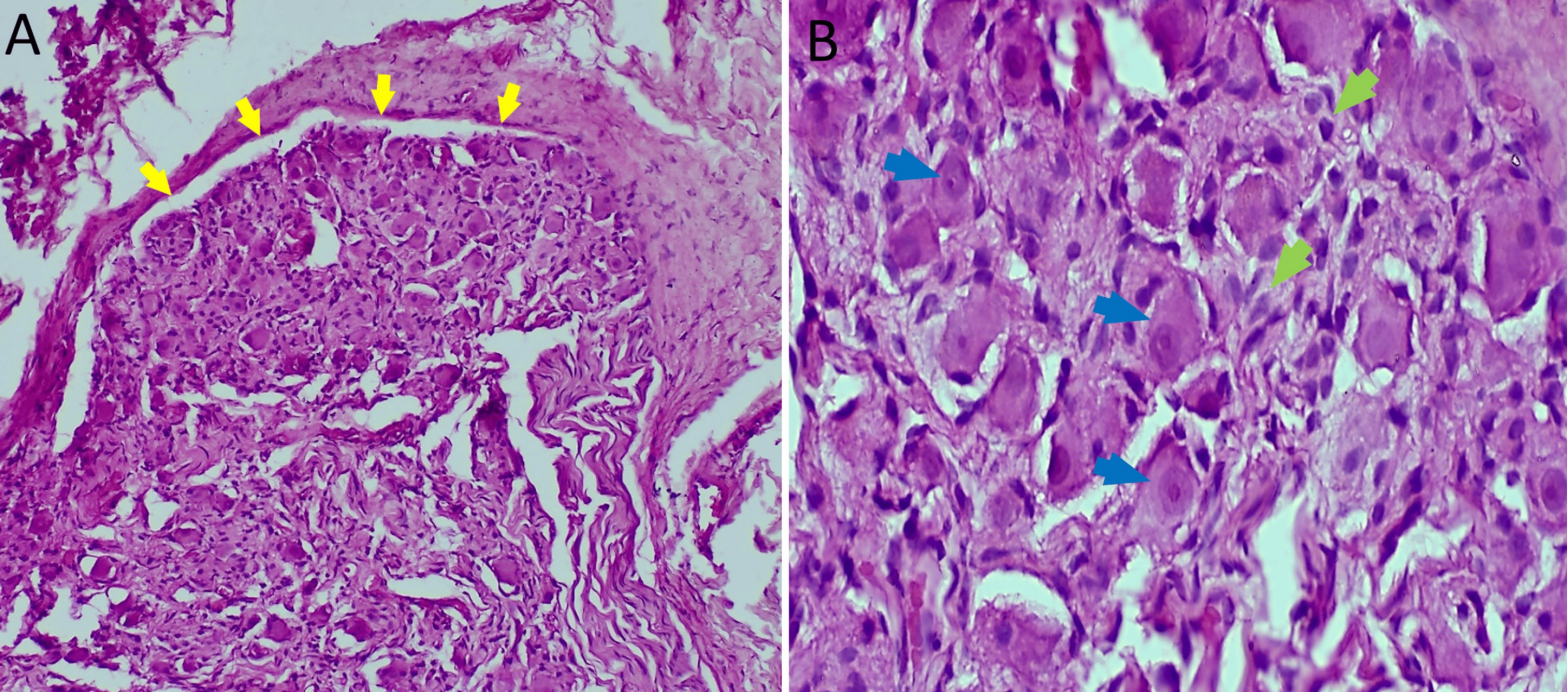
Low-power view (H&E, ×20) showing a well-circumscribed tumor (yellow arrows) composed of mature ganglion cells and spindle cells (A: H&E, ×20). (B) High-power view (H&E, ×40) demonstrating mature ganglion cells with eccentric nuclei, prominent nucleoli, and abundant eosinophilic cytoplasm (blue arrows), interspersed with spindle-shaped Schwann cells (green arrows). H&E: hematoxylin and eosin

Fine needle aspiration cytology (FNAC) revealed normal thyroid follicular cells without cytonuclear atypia or evidence of malignancy. The tumor was completely excised under general anesthesia (Figure 3). Gross examination showed a well-encapsulated, homogeneous, grayish-white mass without areas of hemorrhage or necrosis. Histological analysis revealed a benign, encapsulated tumor composed of clusters of mature ganglion cells with abundant cytoplasm, eccentric nuclei, and prominent nucleoli. The stroma consisted of regular Schwann cells arranged in interlacing fascicles. Extensive sampling revealed no immature neuroblastic components, effectively excluding the possibility of a ganglioneuroblastoma (Figure 4A, 4B).

At two-month follow-up period, the patient demonstrated complete recovery, with no clinical signs of Horner’s syndrome or any other postoperative complications.

## Discussion

Cervical ganglioneuroma is an uncommon, benign neurogenic tumor, accounting for approximately 6% of pediatric tumors [5]. This condition was first described by Loretz in 1870 [6]. Peripheral neuroblastic tumors are classified into four primary subtypes according to the International Neuroblastoma Pathology Classification (INPC): neuroblastoma, ganglioneuroblastoma intermixed, ganglioneuroblastoma nodular, and ganglioneuroma. These categories represent a continuum from the least to the most differentiated forms, assisting in predicting prognosis and guiding treatment decisions [7]. Malignant potential is usually associated with the presence of undifferentiated components; in contrast, ganglioneuromas are composed entirely of mature ganglion cells and Schwannian stroma, reflecting their benign nature [1]. A definitive diagnosis can only be made through histopathological examination.

Ganglioneuromas are typically diagnosed at an older age compared to other peripheral neuroblastic tumors. The incidence ratio of neuroblastoma to ganglioneuroma can be as high as 10:1 [8]. Most ganglioneuromas present as asymptomatic masses in the cervical region. However, symptoms may occur due to compression of nearby anatomical structures. Common clinical signs include difficulty swallowing, shortness of breath, changes in voice, and neck pain. In the reported case, the patient presented with significant dysphonia and contralateral vocal cord paralysis relative to the tumor site [9]. Other possible symptoms include features of Horner’s syndrome, such as ptosis, miosis, anhidrosis, and facial flushing, which typically result from injury to the cervical sympathetic chain. In some cases, systemic effects like hypertension, excessive sweating, diarrhea, and metabolic disturbances such as renal acidosis may develop due to catecholamine secretion, which can increase levels of vanillylmandelic acid (VMA) or homovanillic acid (HVA) [10]. In our patient, no hormonal elevation was observed.

CT imaging usually shows a well-circumscribed, encapsulated mass, with calcifications present in nearly half of the cases, though no calcifications were detected in our case. While imaging can aid in narrowing the differential diagnosis, it is not sufficient to distinguish ganglioneuroma from neuroblastoma or ganglioneuroblastoma definitively. Nonetheless, ganglioneuromas tend to appear more homogeneous on imaging [11].

Surgical excision remains the treatment of choice [1]. The surgical approach depends on the tumor’s size and anatomical location and may include transoral, transparotid, transcervical, or transpharyngeal routes. Surgery is performed both to confirm the diagnosis and to relieve symptoms caused by compression of surrounding structures. These tumors are generally nonaggressive, and even if complete resection is not achieved, recurrence is uncommon. However, surgery in sensitive regions may lead to complications, including nerve or vascular injury, which can sometimes result in Horner’s syndrome [12]. The overall prognosis for patients with ganglioneuroma is favorable, as these tumors do not possess metastatic potential [13].

## Conclusions

Ganglioneuromas are uncommon benign tumors of neuroblastic origin, representing the most mature form in the peripheral neuroblastic tumor classification. While they are typically indolent and asymptomatic, their clinical impact depends on anatomical location and size. Cervical ganglioneuromas are particularly rare and can present with variable symptoms due to local compression, including neurological deficits. Our case illustrates an unusual manifestation of cervical ganglioneuroma with contralateral vocal cord paralysis in a young patient, emphasizing the need for thorough clinical and radiological assessment, followed by histological confirmation. This case contributes to the literature on cervical ganglioneuromas and reinforces the importance of maintaining a broad differential when evaluating neck masses in adolescents.
